# The Combined Contribution of Fear and Perceived Danger of COVID-19 and Metacognitions to Anxiety Levels during the COVID-19 Pandemic

**DOI:** 10.1007/s10942-021-00429-9

**Published:** 2021-11-06

**Authors:** Orkun Aydın, Kuzeymen Balıkçı, Yasin Arslan, Pınar Ünal-Aydın, Ece Müezzin, Marcantonio M. Spada

**Affiliations:** 1 Faculty of Arts and Social Sciences, Department of Psychology, International University of Sarajevo, Sarajevo, Bosnia and Herzegovina; 2 Faculty of Science and Literature, Department of Psychology, International Final University, Nicosia, Turkish Republic of Northern Cyprus; 3 Division of Psychology, School of Applied Sciences, London South Bank University, London, UK

**Keywords:** Anxiety, COVID-19, Danger, Fear, Metacognition

## Abstract

Despite a wide base of research suggesting a major role for dysfunctional metacognitions in contributing to anxiety, their role in explaining psychological distress in the context of the COVID-19 pandemic remains unclear. In this study we investigated whether metacognitions would predict anxiety, while controlling for fear and perceived danger of COVID-19. A total of 862 individuals were included in this study. Participants completed sociodemographic questions, emotional state questions relating to COVID-19, the Metacognitions Questionnaire‐30, and the Generalized Anxiety Disorder-7. Results showed that both negative beliefs about thoughts concerning uncontrollability and danger, and cognitive self-consciousness were significant predictors of anxiety beyond the fear and perceived danger of COVID-19. Future studies involving clinical populations are needed to investigate the longer-term impact of metacognitions in the maintenance and exacerbation of anxiety associated with the fear and perceived danger of COVID-19.

## Introduction

A new coronavirus called SARS-CoV-2 by the Coronavirus Working Group (CSG) (Gorbalenya et al., 2020) was reported in China in December 2019 (Zhu et al., 2020). On February 11, 2020, the disease caused by the new coronavirus was officially named COVID-19 by the World Health Organization (WHO). Evidence has shown that COVID-19 is a different clone from beta-corona viruses associated with the human Severe Acute Respiratory Syndrome (SARS) and the Middle East Respiratory Syndrome (MERS) (Zhu et al., 2020). COVID-19 has become a global emergency state of health in just a few weeks with its unique transmission rate (Wang et al., 2020a). In addition, the number of confirmed and suspected cases has increased rapidly not only in China but also in other countries around the world (Munster et al., 2020). According to reports from the WHO, there were more than 219 million confirmed cases to October 16, 2021 around the globe, of which more than 4.5 million resulted in death. The outbreak has so far been reported in 218 countries and territories outside of China. Previous studies emphasized that psychological disturbance, namely post-traumatic stress disorder, generalized anxiety disorder and depressive disorder may increase in society during and after the major outbreaks (e.g. SARS, MERS) (Mak et al., 2009; Paek et al., 2016). Recent evidence has shown this to be the case with the detrimental psychological effects of the COVID-19 outbreak found to include anxiety, depression (Nikčević & Spada, 2020) somatization, psychological distress (Salazar de Pablo et al., 2020), sleep disturbances (Xiao et al., 2020), suicidal thoughts (Mamun et al., 2020), and post-traumatic stress symptoms (Bo et al., 2020).

In addition, several studies have emphasized the emergence of fear specific to COVID-19 (Ahorsu et al., 2020; Asmundson & Taylor, 2020). Despite fear can be acknowledged as a habitual response to crisis situations, occasionally this response may escalate into excessive anxiety and worry during global life-threatening situations (Haig-Ferguson et al., 2020). Mertens (2020) identified several factors that may elevate the fear of COVID-19 including perceived risks to loved ones, health anxiety, and social media use. Recent studies have also shown that the perceived danger of COVID-19 may escalate stress (Sica et al., 2021; Wang et al., 2020b) and will vary according to contextual differences, including age, gender, and community size (Kimhi et al., 2020). The impact of psychological distress on perceived danger towards adversity among may be particularly marked in youth populations (Braun-Lewensohn & Al-Sayed, 2018; Kimhi et al., 2017).

The effects of fear and perceived danger of COVID-19 on numerous domains on psychological distress appear to be wider than expected with faster transmission of information across the globe (Goyal et al., 2020; Lee et al., 2020; Sica et al., 2021; Wang et al., 2020b; Yıldırım et al., 2020). These studies clearly point to the fact that the fear and perceived danger of COVID-19 are co-occurring with anxiety, but the mechanisms linking these constructs remain unclear.

### The Metacognitive Model of Psychopathology and Its Relevance in Understanding Psychological Distress During the COVID-19 Pandemic

According to the metacognitive model of psychopathology proposed by Wells and Matthews (1996) psychological distress can be escalated by the presence of metacognitions. Metacognitions refer to beliefs we hold about the meaning of internal experiences and how these experiences should be controlled. Positive metacognitions (“If I worry, I will be prepared”) are linked to the initiation of maladaptive coping strategies (such as worry, rumination and thought suppression). Negative metacognitions reflect judgements about the uncontrollability and danger of coping strategies and the consequences of adopting such strategies (e.g. loss of control over thinking, lack of cognitive confidence) (Corcoran & Segal, 2008). Consequently, the presence of metacognitions (and associated maladaptive coping strategies) can lead to an aggravation of distress (Wells, 2008). Previous studies have shown the role of metacognitions in numerous mental health disorders such as anxiety disorders, major depression, schizophrenia, and addictive behaviours (Aydin et al., 2016; Aydın et al., 2019; Papageorgiou & Wells, 2001; Spada et al., 2015). In a similar vein, researchers have demonstrated that metacognitions are associated with an increase in perceived stress as well as the negative affect (Spada et al., 2008b). Additionally, metacognitions may cause and maintain stress through the perseveration of worry (Cook et al., 2015) and may adversely affect the perception of illness (Purewal & Fisher, 2018).

Research has suggested that COVID-19 guidelines (e.g., handwashing) and excessive media exposure may have worsened OCD-type symptoms among adolescents and children (Tanir et al., 2020). Repetitive thoughts on this infectious disease have been found to elevate anxiety and risk of developing trauma (Skalski et al., 2020). However, these studies only measured anxiety levels as a ‘manifestation’ of the impact of COVID-19 pandemic. It still remains unclear which type of beliefs and thinking patterns may play a role in these changes in psychological distress. It is well-known that dysfunctional metacognitions and associated patterns of coping may play a significant role in a wide range of psychopathological presentations (Cartwright-Hatton et al., 2004; Laghi et al., 2018; Mazloom et al., 2016; Moneta, 2011) with recent research supporting the notion that metacognitions may shape one’s negative emotions and psychological distress symptoms (Capobianco et al., 2019).

Previous research has demonstrated the potential relationship between metacognitions and health anxiety symptoms (Melli et al., 2016, 2018), additionally, metacognitions have been found to be a potential mediator of behaviours relating to the fear of COVID-19 (Hashemi et al., 2020). Moreover, it was reported that higher perceived dangerousness and/or susceptibility (i.e. perceived probability of perishing from and/or contracting COVID-19) is associated with higher stress and anxiety levels (Sica et al., 2021; Wang et al., 2020a). Therefore, we postulate that there may be a potential relationship between fear and perceived danger of COVID-19, metacognitions, and anxiety. However, these links have not been explored during the COVID-19 outbreak. In this study we attempt to discern the possible contribution of metacognitions to anxiety, while controlling for fear and perceived danger of COVID-19. We hypothesized that metacognitions would be associated to anxiety whilst controlling for fear and perceived danger of COVID-19. We believe that the possible identification of metacognitions as an independent predictor of anxiety may help focus on essential psychotherapeutic interventions to alleviate anxiety levels during the COVID-19 pandemic.

## Method

### Participants

A virtual snowball (chain) sampling method was used for the study. We placed an advertisement on the Internet and made an announcement through social media platforms for participant recruitment in Turkey and Turkish Republic of Northern Cyprus (TRNC). The participants’ responses were thus collected through online questionnaires. The participants did not receive any incentive or allowance. The single inclusion criterion was to be at least 18 years of age. The single exclusion criterion was to be currently diagnosed and suffering from any form of psychiatric disorder. A total of 1011 individuals participated in the study with 82 participants failing to complete one or more questionnaires. Therefore, statistical analyses were performed with 929 participants. All participants provided consent to participate in the study. The study was approved by the Department of Psychology, International University of Sarajevo (reference no: KISBU/EK/2020/003).

### Measures

#### Metacognitions Questionnaire 30 (MCQ-30; Wells & Cartwright-Hatton, 2004; Tosun & Irak, 2008)

The MCQ-30 is a self-report measure to assess metacognitions. The MCQ-30 consists of 30 items split into five factors: (i) positive beliefs about worry (e.g., “Worrying helps me to get things sorted out in my mind”); (ii) negative beliefs about thoughts concerning danger and uncontrollability (e.g., “My worrying could make me go mad”); (iii) beliefs about the need to control thoughts (e.g., “I should be in control of my thoughts all of the time”); (iv) cognitive confidence (e.g., “I have little confidence in my memory for words and names”); and (v) cognitive self-consciousness (e.g., “I am constantly aware of my thinking”). The MCQ-30 is scored on a 4-point Likert scale (“Do not agree” to “Agree very much”). The total score ranges from 30 to 120, with higher scores reflecting higher levels of maladaptive metacognitions. The MCQ-30 has demonstrated good internal consistency and convergent validity and has acceptable test–retest reliability (Spada et al., 2008a; Wells & Cartwright-Hatton, 2004). The Turkish version of MCQ-30 was used in present study (Cronbach’s α = 0.86) (Tosun & Irak, 2008). Cronbach’s α was found to be 0.91 in our study.

### Generalized Anxiety Disorder-7 (GAD-7; Spitzer et al., 2006; Konkan et al., 2013)

The GAD-7 is a self-report measure to assess anxiety. The GAD-7 consists of seven items assessing anxiety symptoms in the past two weeks. The GAD-7 is scored on 4-point Likert scale scored from zero to three (0 = not at all, 1 = several days, 2 = more than half the days, 3 = nearly every day). Three levels of anxiety are identified: (i) 5–9 as mild; (ii) 10–14 as moderate; and (iii) 15+ severe. The total score ranges from 0 to 21, with higher scores reflecting higher levels of anxiety. The Turkish version of GAD-7 was used in present study (Cronbach’s α = 0.85) (Konkan et al., 2013). Cronbach’s α was found to be 0.90 in our study.

### Sociodemographic and Emotional State Questions Relating to COVID-19

A form including questions about participants’ age, gender, academic qualifications, and marital status was developed by researchers. The form also presented questions regarding levels of fear and perceived danger of COVID-19 (i.e., “What is your fear of the COVID-19 disease?” and “How do you rate the danger of the COVID-19 disease?”). Participants were required to rate their responses from 0 to 7. Higher scores indicated higher levels of fear and perceived danger of COVID-19.

### Statistical Analysis

Normality was checked through skewness, kurtosis, and Shapiro–Wilk testing. All variables were found to be normally distributed across the sample. Sociodemographic data and the descriptive statistics for the responses provided to the emotional state questions relating to COVID-19, the MCQ-30 and the GAD-7 are presented in Tables 1 and 2, respectively.

**Table 1:** Sociodemographic characteristics of the participants

	*n*	*%*	*M*	*SD*
*Age (in years)*			29.6	9.40
*Gender*				
Male	215	22.6		
Female	714	77.4		
*Marital status*				
Single	519	55.9		
Married	410	44.1		
*Academic qualification*				
Literate	1	0.1		
Primary education (8 years of schooling)	15	1.6		
High school	84	9.0		
Bachelor degree or above	828	89.1

**Table 2:** Mean values of MCQ-30 factors, GAD-7, and the participants’ responses to questions relating to fear and perceived danger of COVID-19

	*n*	*%*	*M*	*SD*
MCQ-30-Positive Beliefs about Worry	929		11.8	4.32
MCQ-30-Negative Beliefs about Thoughts Concerning Uncontrollability and Danger	929		13.6	4.56
MCQ-30-Beliefs about the Need to Control Thoughts	929		11.8	4.07
MCQ-30-Cognitive Confidence	929		13.3	4.54
MCQ-30-Cognitive Self-consciousness	929		16.1	4.08
GAD-7	929		5.17	4.84
*What is your fear of the COVID-19 disease?*				
None	90	9.7		
Minor	92	9.9		
Very low	113	12.2		
Low	175	18.8		
High	158	17.0		
Very high	92	9.9		
Extremely high	209	22.5		
*How do you rate the danger of the COVID-19 disease?*				
Non-existent	11	1.2		
Minor	31	3.3		
Very low	91	9.8		
Low	149	16.0		
High	207	22.3		
Very high	132	14.2		
Extremely high	308	33.2		

To explore the relationship between emotional state questions relating to COVID-19, the MCQ-30 factors and GAD-7, Pearson’s Product-Moment correlational analyses were performed. Along with significant variables detected in the correlational analyses, hierarchical multiple regression was run to inspect the value of MCQ-30 factors in predicting GAD-7 scores while controlling for emotional state questions relating to COVID-19. Age and gender were inserted in the first step to control their effects on GAD-7. Fear of COVID-19, perceived danger of COVID-19, and MCQ-30 factors were entered into the following steps, respectively. Cohen’s *f*^2^ that is seen convenient for calculating the effect size was computed; Cohen’s guidelines: *f*^2^ ≥ 0.02, *f*^2^ ≥ 0.15, and* f*^2^ ≥ 0.35 representing small, medium, and large effect sizes, respectively (Cohen, 1988). The statistical significance (*p*) value was set to 0.05 and all analyses were estimated with the statistical analysis software (IBM Corp., Armonk, NY. SPSS Statistics 22.0.).

## Results

### Sample Characteristics

According to the descriptive statistics, most of our participants were comprised by females. Almost half of the sample was single, and the vast majority had a bachelor’s degree (Table 1). Almost a third of the sample (33.2%) considered COVID-19 to be dangerous with 209 participants reporting they were afraid of the disease. Mean values of the utilized tests are demonstrated in Table 2.

### Correlation Analyses

The correlational analyses revealed that GAD-7 was positively correlated with the fear of COVID-19, the perceived danger of COVID-19, and all factors of the MCQ-30. Age and gender were negatively correlated with GAD-7. The fear of COVID-19 was positively correlated with all the factors of the MCQ-30 with the exception of cognitive self-consciousness. Perceived danger of COVID-19 was positively correlated with negative beliefs about thoughts concerning uncontrollability and danger and cognitive confidence. All correlation data is presented in Table 3.

**Table 3:** Correlation coefficients between fear and perceived danger of COVID-19, MCQ-30 factors, and GAD-7

		1	2	3	4	5	6	7	8	9	10	11	12
1	Age												
2	Gender	0.01											
3	Marital Status	− 0.41**	0.16**										
4	Ac. Qual	− 0.09**	− 0.15**	0.08*									
5	Fear of COVID-19	0.06	− 0.12**	− 0.15**	0.00								
6	Perceived Danger of COVID-19	0.05	− 0.09**	− 0.08*	− 0.01	0.61**							
7	MCQ-30-PBW	− 0.03	0.04	0.10**	0.00	0.09**	0.06						
8	MCQ-30-NBW	− 0.11**	− 0.12**	0.03	0.03	0.20**	0.13**	0.34**					
9	MCQ-30-NCT	− 0.11**	0.01	0.06	− 0.03	0.10**	0.05	0.42**	0.62**				
10	MCQ-30-CC	0.08*	− 0.10**	− 0.08*	0.00	0.11**	0.07*	0.32**	0.42**	0.43**			
11	MCQ-30-CSC	− 0.09**	− 0.03	0.05	0.05	0.05	0.04	0.39**	0.58**	0.63**	0.27**		
12	GAD-7	− 0.14**	− 0.08**	0.06	− 0.02	0.33**	0.23**	0.13**	0.51**	0.27**	0.18**	0.19**	0.23**

*n* = 929; Ac. Qual.: Highest academic qualification; MCQ-30-PBW: Metacognitions Questionnaire 30-Positive Beliefs about Worry; MCQ-30-NBT: Metacognitions Questionnaire 30-Negative Beliefs about Thoughts Concerning Uncontrollability and Danger; MCQ-30-NCT: Metacognitions Questionnaire 30-Beliefs about the Need to Control Thoughts; MCQ-30-CC: Metacognitions Questionnaire 30-Cognitive Confidence; MCQ-30-CSC: Metacognitions Questionnaire 30-Cognitive Self-consciousness; GAD-7: General Anxiety Disorder-7. **p < 0.01; *p < 0.05

### Hierarchical Multiple Regression Analysis

The results of the hierarchical multiple regression analysis are shown in Table 4. In the first step, age and gender were significant predictors (F(2, 925) = 11.89, *p* < 0.01) of GAD-7 scores, and accounted for 3% of the variance. In the second step, the fear of COVID-19 contributed significantly to GAD-7 scores, and accounted for an increase to 10% of the variance explained (F(3, 924) = 46.25, *p* < 0.01). In the third step, perceived danger of COVID-19 was not a significant predictor of GAD-7, consequently the explained variance remained unchanged. In the fourth step, negative beliefs about thoughts concerning uncontrollability and danger, and cognitive self-consciousness were significant predictors of GAD-7 scores, accounting for an increase to 20% of the variance explained (F(9, 918) = 50.52, *p* < 0.01). The overall model effect size was large (*f*^2^ ≥ 0.35).

**Table 4:** Hierarchical regression analysis with the fear and perceived danger of COVID-19 and MCQ-30 factors as predictors of GAD-7

	Predictor	B	SE	Beta (*b*)	t	*p*	*R*^*2*^
Step 1							0.03
	Age	− 0.01	0.00	− 0.13	− 4.12	**0.00**	
	Gender	− 0.17	0.07	− 0.08	− 2.54	**0.01**	
Step 2							0.13
	Age	− 0.01	0.00	− 0.15	− 5.00	**0.00**	
	Gender	− 0.09	0.06	− 0.04	− 1.38	0.17	
	Fear of COVID-19	0.14	0.01	0.33	10.59	**0.00**	
Step 3							0.13
	Age	− 0.01	0.00	− 0.16	− 5.03	**0.00**	
	Gender	− 0.09	0.06	− 0.04	− 1.36	0.18	
	Fear of COVID-19	0.13	0.02	0.30	7.57	**0.00**	
	Perceived Danger of COVID-19	0.03	0.02	0.05	1.37	0.17	
Step 4							0.33
	Age	− 0.01	0.00	− 0.10	− 3.62	**0.00**	
	Gender	0.01	0.06	0.01	0.26	0.79	
	Fear of COVID-19	0.09	0.02	0.21	6.11	**0.00**	
	Perceived Danger of COVID-19	0.02	0.02	0.04	1.21	0.23	
	MCQ-30-PBW	0.00	0.01	− 0.02	− 0.65	0.51	
	MCQ-30-NBT	0.10	0.01	0.54	13.94	**0.00**	
	MCQ-30-NCT	0.00	0.01	0.00	0.05	0.96	
	MCQ-30-CC	0.00	0.01	− 0.02	− 0.72	0.47	
	MCQ-30-CSC	− 0.03	0.01	− 0.12	− 3.29	**0.00**	

*n* = 929; MCQ-30-PBW: Metacognitions Questionnaire 30-Positive Beliefs about Worry; MCQ-30-NBT: Metacognitions Questionnaire 30-Negative Beliefs about Thoughts Concerning Uncontrollability and Danger; MCQ-30-NCT: Metacognitions Questionnaire 30-Beliefs about the Need to Control Thoughts; MCQ-30-CC: Metacognitions Questionnaire 30-Cognitive Confidence; MCQ-30-CSC: Metacognitions Questionnaire 30-Cognitive Self-consciousness. Significant observations are highlighted in bold

## Discussion

The aim of the present study was to investigate to what extent metacognitions may contribute to anxiety when fear and perceived danger of COVID-19 are controlled for. Despite the detrimental effects of the perceptions towards the COVID-19 outbreak, the result of the multiple regression analysis showed that both negative beliefs about thoughts concerning uncontrollability and danger, and cognitive self-consciousness were significant predictors of anxiety above and beyond the fear and perceived danger of COVID-19.

How can we explain these findings within a metacognitive paradigm? Negative beliefs about thoughts concerning uncontrollability and danger are a marker of the activation of repetitive negative thinking patterns (e.g., worry) as a means of coping. These beliefs have been found to be strong predictors of generalized anxiety disorder (Wells, 1995; Wells & Carter, 2001). Previous research has also highlighted the impact of COVID-19 based on perceived risk, anxiety and depression levels (Choi et al., 2020; Hyland et al., 2020; Khademian et al., 2021; Moghanibashi-Mansourieh, 2020) which are closely linked to worry and other forms of maladaptive coping. Beliefs about thoughts concerning uncontrollability and danger may bring to increased levels of hypersensitiveness to fear and perceived danger of COVID-19, leading to the development of corresponding behavioral routines like self-constraints towards any form of exposure to media coverage or news relating to COVID-19, avoidance of social engagement, threat monitoring, etc. which may escalate anxiety and further confirm a sense of uncontrollability and danger (Nikčević & Spada, 2020; Nikčević et al., 2021).

Turning now to the findings on cognitive self-consciousness, the second metacognition found to independently predict higher anxiety levels, it is plausible to assume that individuals who report paying close attention to their thoughts/engaging in excessive self-monitoring will observe/notice more fear and perceived danger of COVID-19. In line with this view, Hatabu et al. (2020) argued that individuals with higher scores in private self-consciousness tend to take strict protection measures to address the COVID-19 guidelines. A possible reason underlying this behavior attitude may be explained by excessive cognitive monitoring which triggers individuals to obey the guidelines to get relief from anxiety. In other words, individuals may attempt to regulate fear and perceived danger of COVID-19 by cognitive reframing (e.g., “I should be careful about COVID-19”) which may then evolve into heightened cognitive self-consciousness (e.g., “I think a lot about, and notice, my thoughts and fears about COVID-19”) which may, in turn, be accompanied by negative beliefs about thoughts concerning uncontrollability and danger (e.g., “I cannot ignore my worrying thoughts about COVID-19”), thus elevating anxiety levels.

In a recent study conducted by Li et al. (2020) it was suggested that Cognitive Behaviour Therapy (CBT) may be a useful intervention to tackle a wide range of psychopathology during COVID-19. However, typically, the cognitive interventions within CBT only involve challenging thoughts with factual information which may have been the reason behind smaller effects on anxiety symptoms. Wang et al. (2021) have demonstrated that anxiety interventions involving cognitive emotion regulation strategies (rather than the modification of the content of thinking) may be more effective. Furthermore Xu et al. (2020) have also highlighted the importance of wider cognitive interventions that involve one’s perception of stress where individuals may not be *worried about worrying* and see it as a resource to address the guidelines during COVID-19. The principal psychological therapy that focus on the modification of metacognitions and associated control strategies (worry, rumination and thought suppression) is Metacognitive Therapy (Wells, 2013). A wide evidence base has shown that metacognitive interventions such as detached mindfulness, attention training and the restructuring of metacognitions may bring to significant improvements in psychological distress (Normann et al., 2014). It is plausible to assume that this form of psychological therapy may benefit individuals who are trapped in self-referent forms of coping (worry, rumination, etc.) about fears and perceived danger of COVID-19.

It should also be noted, that both Turkish and TRNC governments have been applying several significant precautions including prohibition of crowded gatherings, closing public facilities, restricting public transportation, requiring self-isolation, closing country borders, suspension of flights to/from risky countries to limit the spread of the COVID-19 outbreak. However, each of these interventions may also affect the psychological well-being of the society and there is still gap in studies focusing on effective targets to alleviate the psychological disturbances during COVID-19 pandemic. Understanding the role of metacognitions and associated forms of coping in the escalation of COVID-19 psychological distress (fear and danger perception) may be of value going forward.

There are several limitations to our study that need to be noted. The participants’ mental status was not assessed with a structured clinical interview. Therefore, our sample may not thoroughly reflect the non-psychiatric population. The cross-sectional design of the study precludes us to draw strong conclusions regarding causative associations between the study variables. The potential for selection bias should also be considered with web-based survey methodology. Lastly, we have measured the intensity of the emotions and perceptions related to COVID-19 with subjective gradings, hence this may limit our interpretations.

In conclusion, this study investigated the impact of metacognitions on anxiety during the COVID-19 outbreak and attempted to outline how anxiety may be magnified by the presence of metacognitions beyond the fear and perceived danger of COVID-19. In March 2020, WHO declared a wide range of recommendations to overcome mental health issues and they emphasized the potential detrimental effects of the COVID-19 outbreak on psychological well-being (World Health Organization, 2020). Primary issues to be addressed in further studies may involve the impact of interventions that consider a wider focus on cognition including metacognitions.

## Declarations

### Availability of Data and Material

The data will be made available upon request.
